# Oncogenic *FGFR1* mutation and amplification in common cellular origin in a composite tumor with neuroblastoma and pheochromocytoma

**DOI:** 10.1111/cas.15260

**Published:** 2022-02-16

**Authors:** Keiji Tasaka, Hiroo Ueno, Kai Yamasaki, Takahiro Okuno, Tomoya Isobe, Shunsuke Kimura, Katsutsugu Umeda, Junichi Hara, Seishi Ogawa, Junko Takita

**Affiliations:** ^1^ Department of Pediatrics, Graduate School of Medicine Kyoto University Kyoto Japan; ^2^ Department of Pediatric Hematology and Oncology Osaka City General Hospital Osaka Japan; ^3^ Department of Pathology Osaka City General Hospital Osaka Japan; ^4^ Department of Pediatrics, Graduate School of Medicine The University of Tokyo Tokyo Japan; ^5^ Department of Pediatrics Hiroshima University Graduate School of Biomedical Sciences Hiroshima Japan; ^6^ Cancer Consultation and Support Center Osaka City General Hospital Osaka Japan; ^7^ Department of Pathology and Tumor Biology, Graduate School of Medicine Kyoto University Kyoto Japan; ^8^ Institute for the Advanced Study of Human Biology (WPI‐ASHBi) Kyoto University Kyoto Japan; ^9^ Department of Medicine, Center for Hematology and Regenerative Medicine Karolinska Institute Stockholm Sweden

**Keywords:** common cellular origin, composite tumor, *FGFR1*, neuroblastoma, pheochromocytoma

## Abstract

Neuroblastoma (NB) and pheochromocytoma (PCC) are derived from neural crest cells (NCCs); however, composite tumors with NB and PCC are rare, and their underlying molecular mechanisms remain unknown. To address this issue, we performed exome and transcriptome sequencing with formalin‐fixed paraffin‐embedded (FFPE) samples from the NB, PCC, and mixed lesions in a patient with a composite tumor. Whole‐exome sequencing revealed that most mutations (80%) were shared by all samples, indicating that NB and PCC evolved from the same clone. Notably, all samples harbored both mutation and focal amplification in the *FGFR1* oncogene, resulting in an extraordinarily high expression, likely to be the main driver of this tumor. Transcriptome sequencing revealed undifferentiated expression profiles for the NB lesions. Considering that a metastatic lesion was also composite, most likely, the primitive founding lesions should differentiate into both NB and PCC. This is the first reported case with composite‐NB and PCC genetically proven to harbor an oncogenic *FGFR1* alteration of a common cellular origin.

## Graphical abstract

This is the first reported case with composite tumor with neuroblastoma (NB) and pheochromocytoma (PCC) genetically proven to harbor an oncogenic FGFR1 alteration. NB and PCC shared the same cellular origin and FGFR1 mutation should be a main driver of this tumor. This composite characteristic was not driven by distinct gene mutations. Instead, primitive founding lesions should differentiate into both NB and PCC.

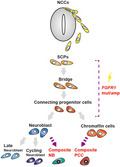

**Abbreviations**
CNA: copy number alteration
CPT‐11: irinotecan
FFPE: formalin‐fixed paraffin‐embedded
MIBG: ^123^I‐metaiodobenzylguanidine
NB: neuroblastoma
NCCs: neural crest cells
PCC: pheochromocytoma
SCPs: Schwann cell precursors
TARGET: Therapeutically Applicable Research to Generate Effective Treatment
TMZ: temozolomide
VAFs: variant allele frequencies
WES: whole‐exome sequencing

## 1 INTRODUCTION

PCC and NB are the most common NCC‐derived tumors in adults and children, respectively.^1^, ^2^, ^3^ Composite pheochromocytoma refers to tumors with morphologic features of PCC and NCC‐derived tumors, such as malignant peripheral nerve sheath tumor and neuroendocrine carcinomas, within the same tumor.^4^, ^5^ Composite tumors are rare and most often combined with ganglioneuroma in composite PCC; therefore, composite tumors comprising PCC and NB are even rarer.^5^, ^6^, ^7^, ^8^, ^9^, ^10^ The genetic mechanism of composite tumors with PCC and NB remains unclear; a single nucleotide polymorphism array analyzed only 1 case.^5^ Here, we applied exome and transcriptome analyses to a patient case with a composite tumor with NB and PCC to investigate whether the NB and PCC lesions arose from a common cellular origin and how this tumor developed.

## 2 MATERIALS AND METHODS

Detailed methods are provided in the Supporting information section of this paper and include the following:

- Patient samples
- Immunohistochemistry analysis
- Whole‐exome sequencing and mutation calling
- Validation of detected mutations
- Phylogenetic analysis
- Analysis of alterations in copy number
- RNA sequencing and gene expression analysis
- Accuracy of RNA‐seq data generated from FFPE samples

This study was approved by the Institutional Review Board of Kyoto University. Written informed consent was obtained from the patient's parents.

## 3 RESULTS

### 3.1 Case presentation

**FIGURE 1:**
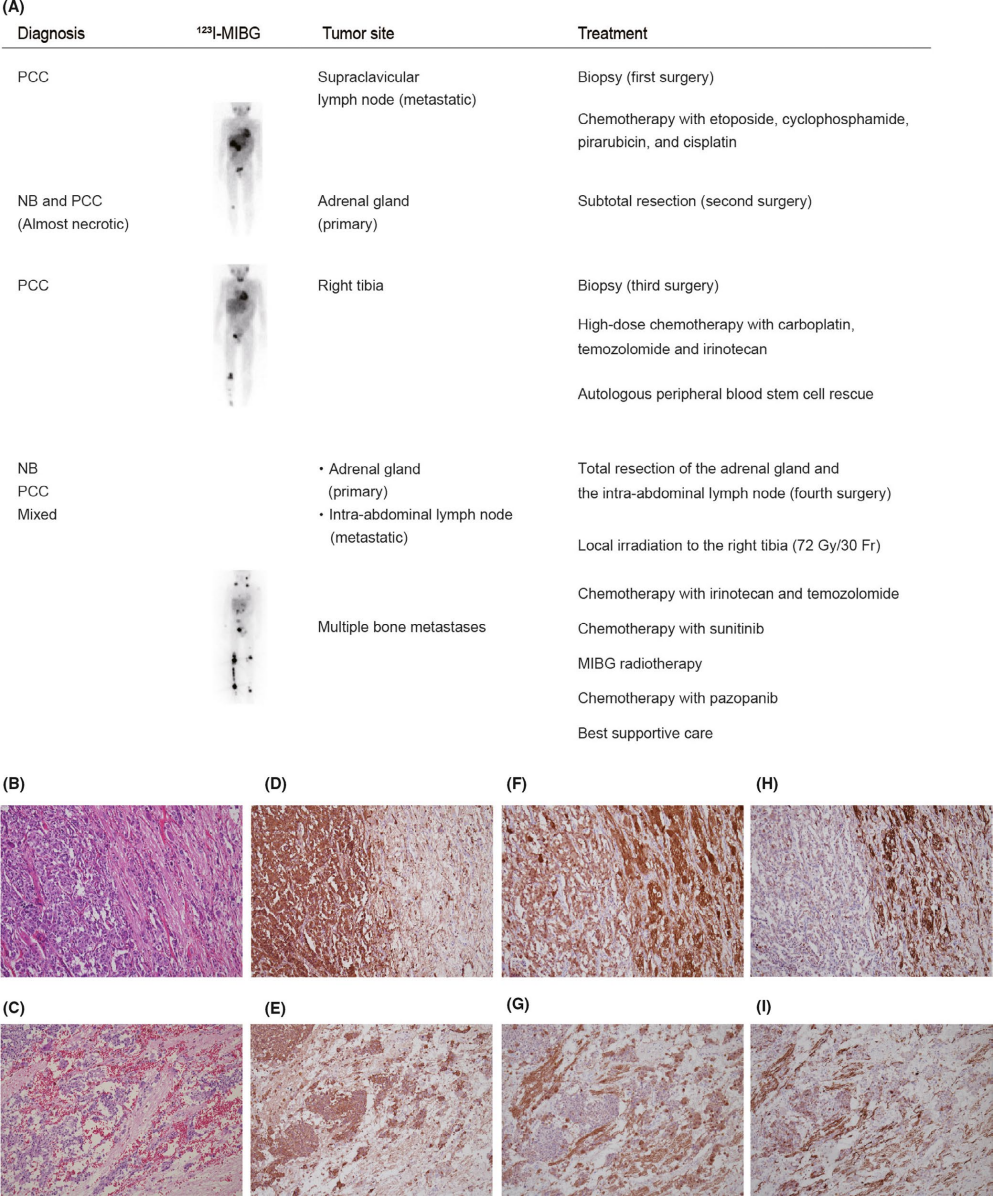
Clinical presentation in this case, and histological features of the resected tumors at the fourth surgery. A, Clinical presentation in this case. B, C, H&E staining of the adrenal primary tumor showing 2 distinct patterns (PCC: left, NB: right) (B) and a mixed pattern (C). D, E, The PCC component is more strongly positive for chromogranin A. F, G, The NB component is more strongly positive for PGP9.5. H, I, Neurofilament is largely restricted to the NB component. Original magnification: ×100 (B–I). NB, neuroblastoma; PCC, pheochromocytoma; mixed, mixed components of NB and PCC without clear boundaries

A 5‐y‐old boy was admitted with abdominal pain. Computed tomography and ^123^I‐metaiodobenzylguanidine (MIBG) scintigraphy revealed lesions in the adrenal gland and supraclavicular lymph node (Figure 1A). A metastatic supraclavicular lymph node biopsy was performed (first surgery), and histopathological tissue assessment verified the diagnosis of metastatic PCC. The patient had no medical history of malignant diseases or a family history of cancer. He underwent 4 cycles of multidrug chemotherapy with etoposide (100 mg/m^2^/d on days 1‐5), cyclophosphamide (1200 mg/m^2^/d, day 1), pirarubicin (40 mg/m^2^/d, day 3), and cisplatin (90 mg/m^2^/d, day 5), followed by partial resection of the primary tumor (second surgery). Most of the resected tumor was necrotic; however, a new metastatic lesion with PCC was found at the proximal end of the patient's right tibia (third surgery). Therefore, the patient underwent high‐dose chemotherapy with carboplatin (500 mg/m^2^/d, days 1, 2), temozolomide (TMZ) (250, days 1‐5), and irinotecan (CPT‐11) (50, days 1‐5), with an autologous peripheral blood stem cell rescue. Then, the primary adrenal gland and metastatic intra‐abdominal lymph node were entirely resected (fourth surgery). Both lesions contained clearly defined areas of NB, PCC, and their mixed components without clear boundaries (Figure 1B‐I). The NB lesions demonstrated a slight response to the treatment, while the PCC lesions were treatment resistant and tended to increase. Multiple bone metastases appeared despite local irradiation of the right tibia (72 Gy/30 Fr) and 2 cycles of CPT‐11 and TMZ. Subsequently, the patient received sunitinib; however, the treatment was quickly terminated due to a rash. He then received MIBG radiotherapy and pazopanib; however, he died from disease progression (Figure 1A).

### 3.2 Composite‐NB and PCC have the same cellular origin

**FIGURE 2:**
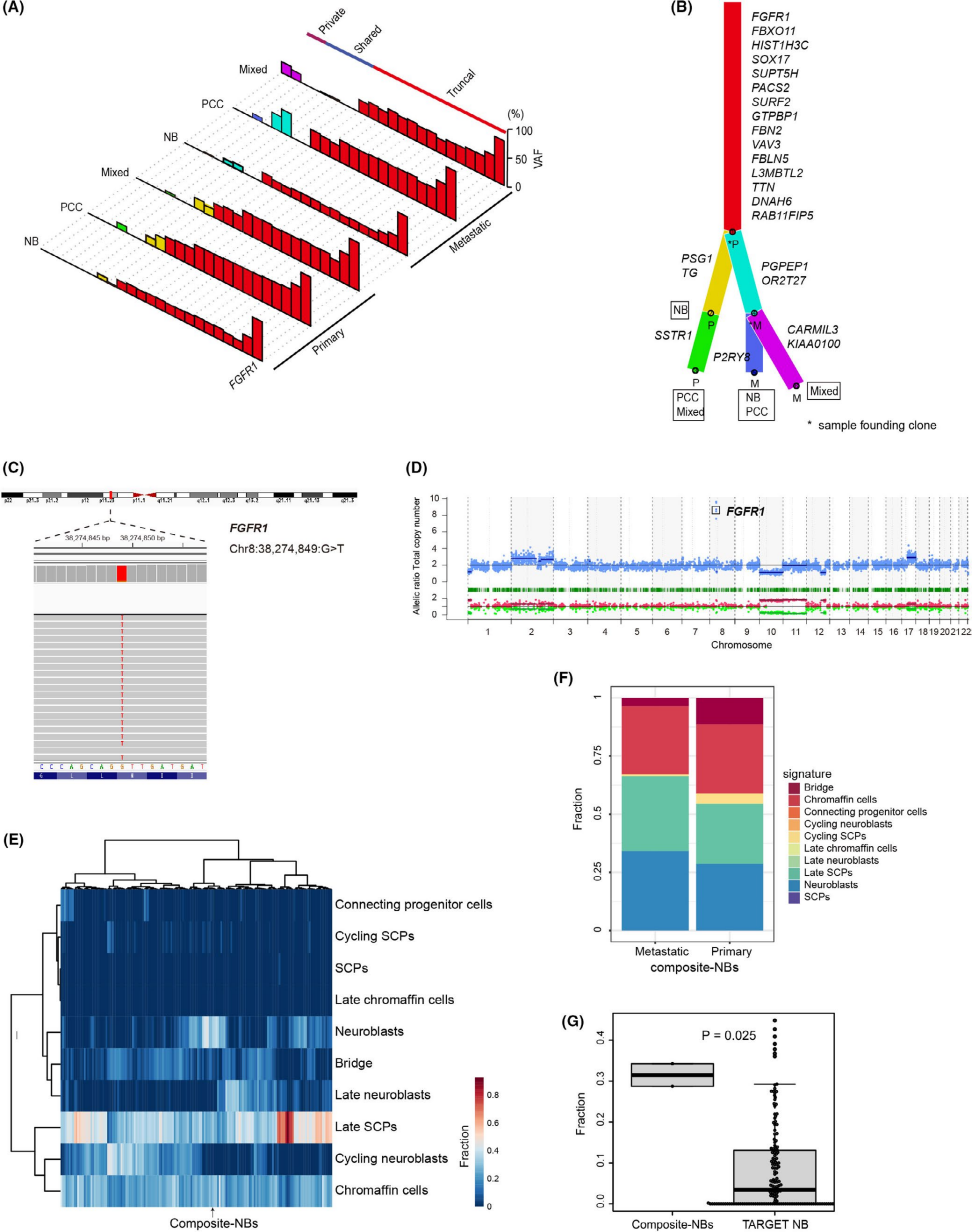
Mutational landscape of the composite tumor, and classification of composite‐NBs and deposited database of NB samples based on expression profiles. A, Landscape of the somatic mutations of each lesion in the primary adrenal gland and metastatic intra‐abdominal lymph node samples. The bars represent variant allele frequencies (VAFs). B, The phylogenetic tree was constructed using ClonEvol based on the mutational landscape. C, Representative sequencing data of the PCC component in the primary adrenal lesion revealed mutation in chr8:38274849:G>T (*FGFR1* N546K). D, Representative copy number plot of the PCC component in the primary adrenal lesion with *FGFR1* amplification. E, Hierarchical clustering of composite‐NBs and TARGET cohort NBs based on the abundance of fetal adrenal medullary cell populations as determined by BSEQ‐sc. F, Composition of composite‐NBs based on the deconvolution of bulk RNA‐seq data with fetal adrenal cell populations using BSEQ‐sc. G, Proportion of neuroblasts in composite‐NBs when compared with the TARGET cohort NBs. The *P*‐value was calculated using the Wilcoxon rank‐sum test. NB, neuroblastoma; PCC, pheochromocytoma; mixed, mixed components of NB and PCC without clear boundaries; SCPs, Schwann cell precursors

Using FFPE samples at the fourth surgery, we extracted DNA and RNA from a total of 6 lesions as follows: 1 from each of the NB, PCC, and mixed lesions in the primary adrenal gland and of the metastatic intra‐abdominal lymph node. We identified somatic mutations and copy number alterations (CNA) in all DNA samples using WES. Consequently, 108 somatic mutations were detected and validated by amplicon deep sequencing (median: 18 per sample; range: 17‐19) (Table S1). Fifteen mutations were found in all 6 samples, indicating NB, PCC, and mixed lesions evolved from the same neoplastic clone (Figure 2A,B). Additionally, there were no NB or PCC‐specific mutations, suggesting the composite characteristic was not driven by distinct gene mutations (Figure 2A,B).

Notably, *FGFR1* N546K, a rare but known cancer‐related somatic mutation in conventional NB^11^ and PCC,^2^ had the largest VAFs and underwent focal amplification in all samples (Figures 2A‐D and S1), implying that this mutation and amplification were one of the main drivers of the tumor. Regarding VAFs, *FGFR1* N546K might have occurred before amplification. No germline mutation was observed in the genes related to familial neuroblastoma and hereditary paraganglioma‐pheochromocytoma syndrome.^12^

According to the sequence‐based CNA analysis, all 6 samples harbored identical CNA profiles of del (1p), +2, del (10), 11UPD, and +17q. Del (7) was observed only in the mixed lesion of the metastatic intra‐abdominal lymph node. Compared with conventional NB and PCC, del (1p) is recurrently in both NB^11^, ^13^ and PCC,^2^ and 11UPD and +17q are only in NB.^11^, ^13^ Del (10) and del (7) are uncharacteristic of both NB and PCC (Figure S1).

### 3.3 Composite‐NBs transcriptionally contain larger fractions of early normal fetal adrenal neuroblasts

We further illustrated the molecular basis of this composite tumor by performing whole‐transcriptome sequencing of the 6 samples. Unsupervised consensus clustering identified 2 clusters completely corresponding to the histopathological features of NB and PCC. One mixed lesion with higher NB components was classified with the NB lesion samples, and the other mixed lesion with higher PCC components was classified with the PCC lesion samples (Figure S2A,B). As the expression profiles of the 2 mixed lesions were heterogeneous and affected by the amount of NB or PCC components, the 4 samples from pure NB (composite‐NBs) and PCC (composite‐PCCs) lesions were used for subsequent analyses.

Next, we analyzed the expression profiles of the composite‐NBs combined with 161 conventional NBs in the TARGET cohort (https://portal.gdc.cancer.gov/projects). Recently, single‐cell transcriptomic analyses of the developmental origins of NB defined normal differentiation trajectories from SCPs over the intermediate states to neuroblasts or chromaffin cells, suggesting that NB transcriptionally resemble normal fetal adrenal neuroblasts.^14^ To analyze the composition and developmental programs in composite‐NBs, we used the expression signatures of normal adrenal medullary cell populations^14^ to decompose the bulk transcriptomes of composite‐NBs and TARGET cohort NBs (Table S2). Most of the NBs, including composite‐NBs, were confirmed to transcriptionally match the normal neuroblasts (Figure 2E). Unexpectedly, in composite‐NBs, only a few late neuroblasts (differentiated neuroblasts) were detected; however, the abundance of neuroblasts (early neuroblasts) was higher than that in TARGET NB cohort (Figure 2F,G), suggesting that composite‐NBs were of transcriptionally undifferentiated subtype. The analysis of the composition between composite‐NBs and composite‐PCCs revealed that composite‐PCCs had a higher population of chromaffin cells and lower populations of neuroblasts when compared with composite‐NBs (Figure S3A).

We compared the expression pattern of composite‐PCCs to that of conventional PCCs by performing the unsupervised consensus clustering of composite‐PCCs and 173 PCC/PGL samples in TCGA.^2^ Expectedly, composite‐PCCs were clustered with the conventional PCCs of a kinase signaling subtype (Figure S3B,C), in which *FGFR1* N546K was recurrent.^2^

### 3.4 Inflammatory pathways are activated in composite‐NBs compared with composite‐PCCs

We illustrated statistically differential signaling pathways between composite‐NBs and PCCs using Gene Set Enrichment Analysis with MSigDB hallmark gene sets.^15^ Compared with the composite‐PCCs, enrichment of inflammatory response pathways and negative enrichment of proliferation‐associated pathways, including E2F targets and G2M checkpoints, were observed in the composite‐NBs (Figure S4A). The transcriptional induction of an IFN response within tumor cells indicates the contribution of host immunity to the therapeutic response^16^; therefore, high inflammatory signals may reflect a contribution of host immunity. In fact, immunohistochemical staining revealed more CD68^+^ macrophages infiltrated into tumor tissues in composite‐NBs than in PCCs (Figure S4B). These results agreed with the clinical course that the composite‐NBs responded to treatment more than the composite‐PCCs.

## 4 DISCUSSION

The whole‐exome and transcriptome sequencing of composite‐NBs and PCCs revealed that the NB and PCC lesions shared a common cellular origin with the *FGFR1* alteration, and that composite‐NBs had undifferentiated features. Although the evolution of this composite tumor remains unclear, this study provided important clues to this question from 3 perspectives.

First, composite‐NB and PCC share the same cellular origin, and the *FGFR1* N546K mutation with focal amplification is likely to be the main driver for this tumor. *FGFR1* is commonly activated through amplification in tumors, such as breast^17^ and lung cancer,^18^ and recurrent *FGFR1* somatic mutations are identified in pilocytic astrocytoma.^19^ Furthermore, the p.Asn546Lys (N546K) variant alters *FGFR1* autophosphorylation, increasing kinase activity, and transforming potential.^20^ Although *FGFR1* mutations have been observed in NB and PCC,^2^, ^11^ we are the first to report the co‐occurrence of mutation and focal amplification in *FGFR1* causing high expression (Figure S5), conferring an aggressive phenotype.

Regarding neural development, fibroblast growth factor signaling plays multiple roles necessary for NCC induction and specification, such as patterning *Hox* expression via *Cdx* genes, posteriorizing neural plate, inducing paraxial mesoderm, inhibiting bone morphogenetic protein (BMP) signaling and BMP expression, and inducing *WNT* gene expression.^21^ Therefore, the *FGFR1* mutation with amplification in differentiating NCC cells is likely to confer growth advantages and contributes to the development of tumor‐initiating cells from which NB and PCC may evolve.

Second, the accumulation of distinct mutations would be irrelevant to the characteristics of this composite tumor. Contrary to myelodysplastic syndromes, in which sequential mutation acquisitions are pivotal for clonal evolution to acute myeloid leukemia,^22^ NB or PCC‐specific mutations were not detected in this composite tumor. In addition, the number of somatic mutations was low, and the mutations and CNAs were mostly shared by the samples. Nevertheless, the accumulation of shared mutations and common CNAs, such as del (1p), 11UPD, and +17q, would contribute to the pathogenesis of this tumor. Del (7) was observed only in the mixed lesion of the metastatic intra‐abdominal lymph node, and not in the other lesions. Therefore, tumors are believed to consist of a heterogeneous mixture of functionally distinct cancer cells and that their subpopulations vary widely in their responses to therapeutic agents.^23^ In the present case, it seems possible that the clone with del (7) was present as a minor clone in other lesions, albeit this clone subsequently became dominant in this mixed lesion. Nevertheless, whether the significance of this del (7) is related to drug resistance or malignancy remains unclear.

Third, the expression profile of composite‐NBs was mainly similar to that of early normal neuroblasts, which suggests that the *FGFR1* alteration may be acquired in less differentiated progenitor cells. This speculation is supported by the fact that, in a mixed phenotype acute leukemia, which is also a composite of lymphoid and myeloid hematopoietic lineages, mutations are acquired in early hematopoietic progenitor cells, which then drives the bi‐phenotypic nature.^24^

**FIGURE 3:**
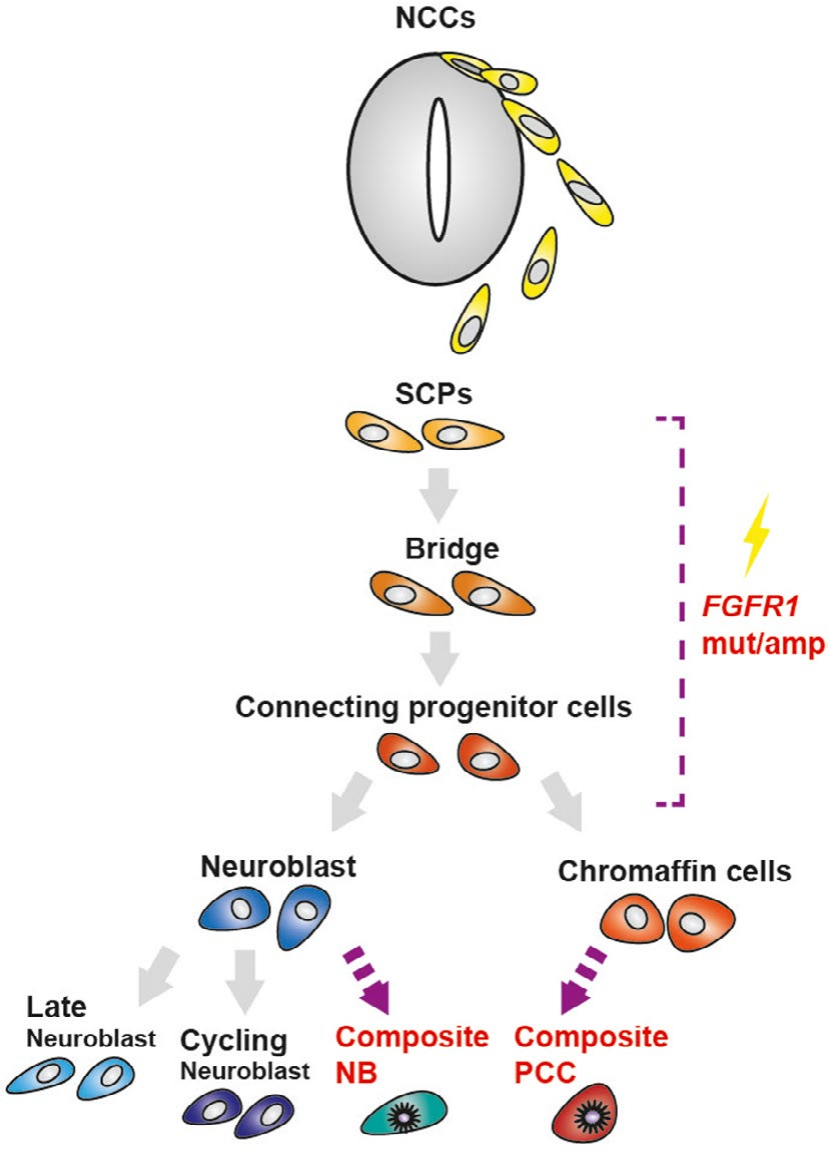
Schematic modeling of composite‐NB/PCC development from SCPs through the acquisition of *FGFR1* alterations along the representation of conventional‐NB identity. NB, neuroblastoma; NCCs, neural crest cells; PCC, pheochromocytoma; mut/amp, mutation, and amplification; SCPs, Schwann cell precursors

To the best of our knowledge, this is the first case with composite‐NB and PCC, genetically proven to harbor a common cellular origin. Furthermore, our results suggest a possible mechanism for the formation of this composite characteristic in line with a previous report^14^ (Figure 3). However, as there were no viable cells left, in vivo validation using xenograft models or cell lines derived from this composite tumor was impossible; therefore, further studies, including those on other composite tumors, are needed to investigate whether the stemness is related to the formation of composite tumors.

## DISCLOSURE

The authors have no conflict of interest.

## Supporting information

Figure S1‐S7

Table S1‐S2

Appendix S1
