# Supplementary material for: Oncogenic FGFR1 mutation and amplification in common cellular origin in a composite tumor with neuroblastoma and pheochromocytoma
Source: Cancer Sci. 2022 Feb 16;113(4):1535–41. doi: 10.1111/cas.15260 (PMC8990717; doi:10.1111/cas.15260)
Supplement: Supplementary file 1 — Figure S1‐S7 [file CAS-113-1535-s001.pdf]

Figure S1

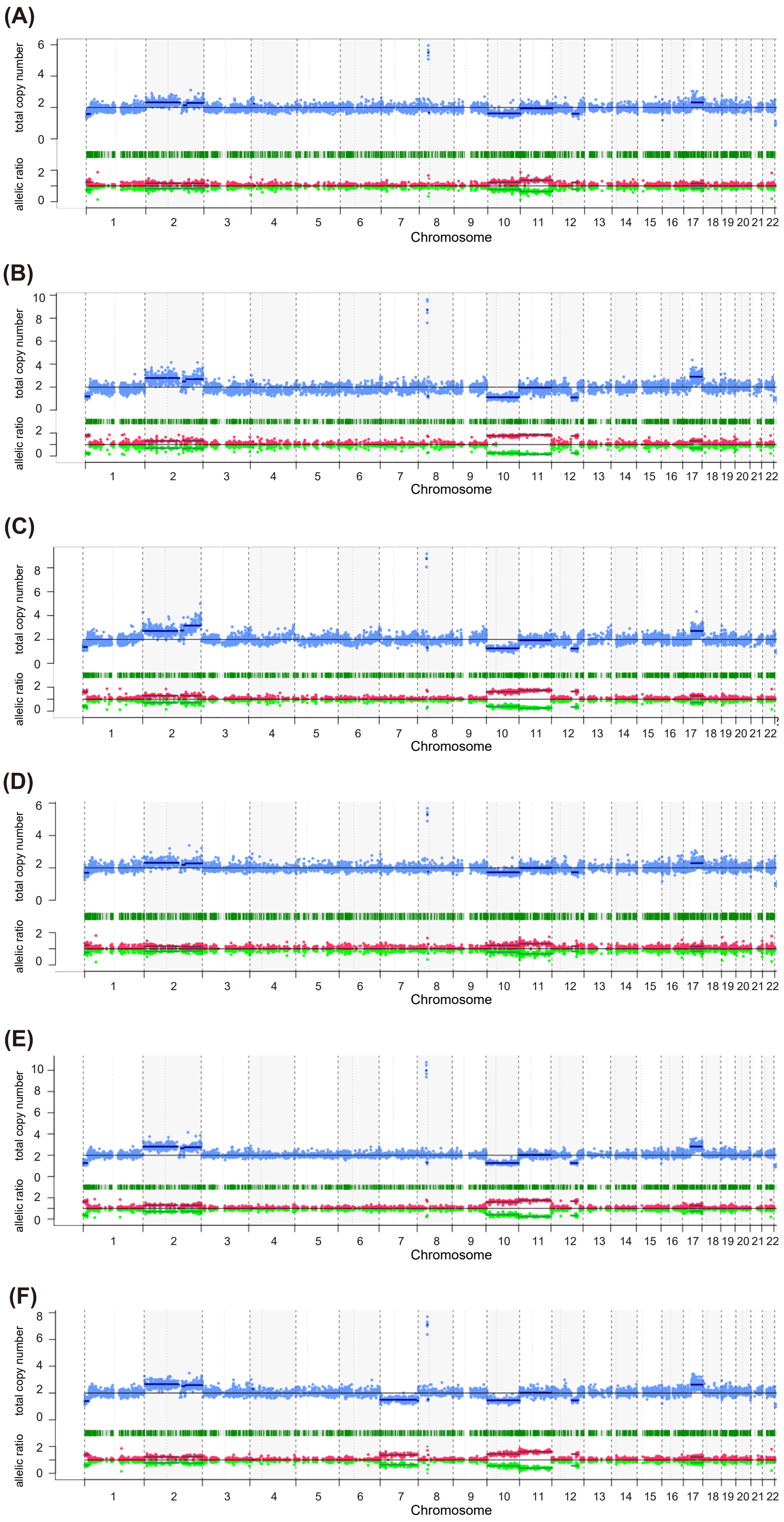

Figure S1. Copy number plots of each of the six samples of the composite tumor.
A, NB lesion in the primary adrenal gland
B, PCC lesion in the primary adrenal gland
C, Mixed lesion in the primary adrenal gland
D, NB lesion in the metastatic intra-abdominal lymph node
E, PCC lesion in the metastatic intra-abdominal lymph node
F, Mixed lesion in the metastatic intra-abdominal lymph node

Figure S2

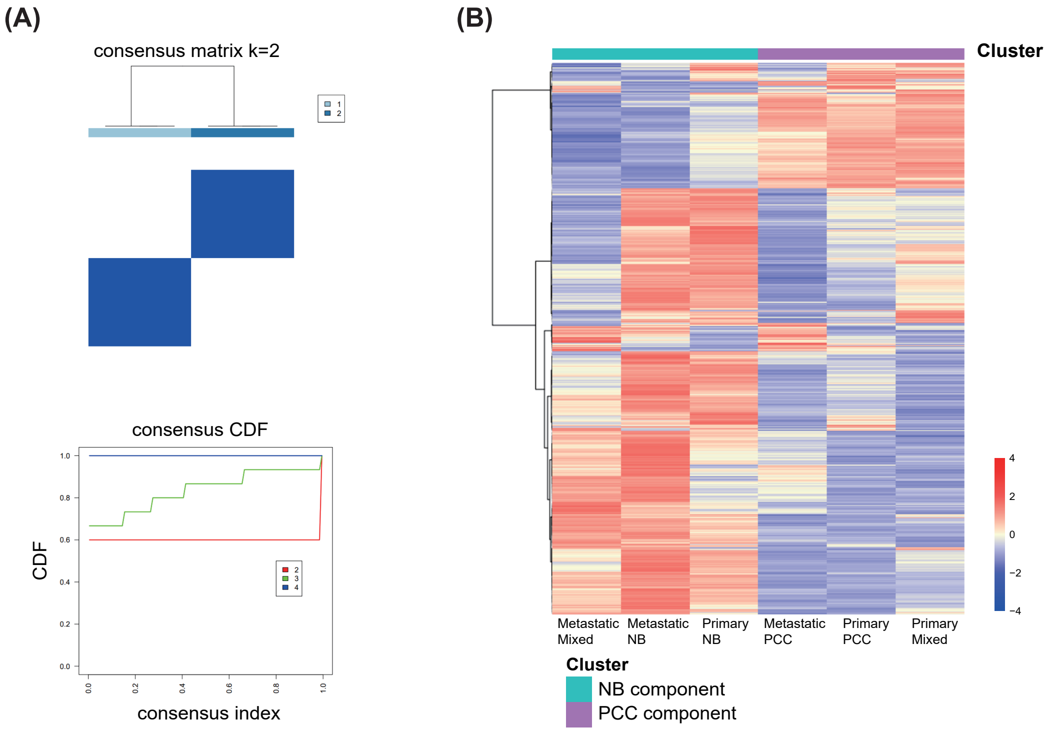

Figure S2. Consensus clustering analysis of the six samples of the composite tumor.
A, CDF plots and consensus matrices for consensus clustering (n = 1,000) of the six samples of the composite tumor. Two thousand differentially expressed genes were selected. B, Unsupervised consensus clustering of the six samples of composite tumor identified two distinct clusters corresponding to histopathological features of NB and PCC.
NB, neuroblastoma; PCC, pheochromocytoma; mixed, mixed components of NB and PCC without clear boundaries.

Figure S3

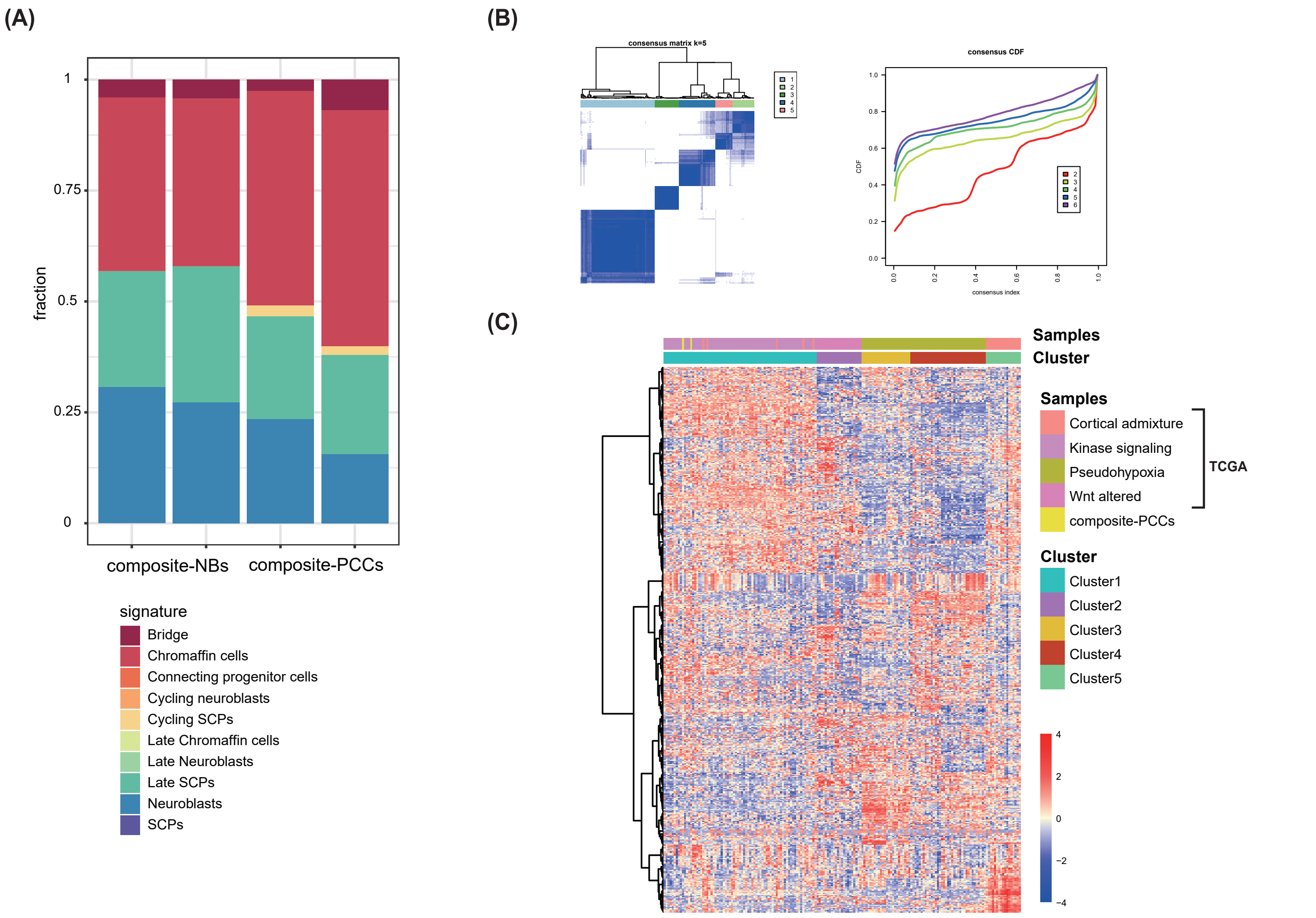

Figure S3. The composition of composite-NBs and PCCs, and the gene expression profiles in composite-PCCs and 173 TCGA PCC/PGL samples based on unsupervised consensus clustering. A, Composition of composite-NBs and PCCs based on deconvolution of bulk RNA sequencing data with fetal adrenal cell populations using BSEQ-sc. B, CDF plots and consensus matrices for consensus clustering (n = 1,000) of two composite-PCCs and 173 TCGA PCC/PGL samples. Five hundred differentially expressed genes were selected. C, A heatmap of the expression data of two composite-PCCs and 173 TCGA PCC/PGL samples. Composite-PCCs were grouped into the same cluster of kinase signaling of TCGA samples.

Figure S4

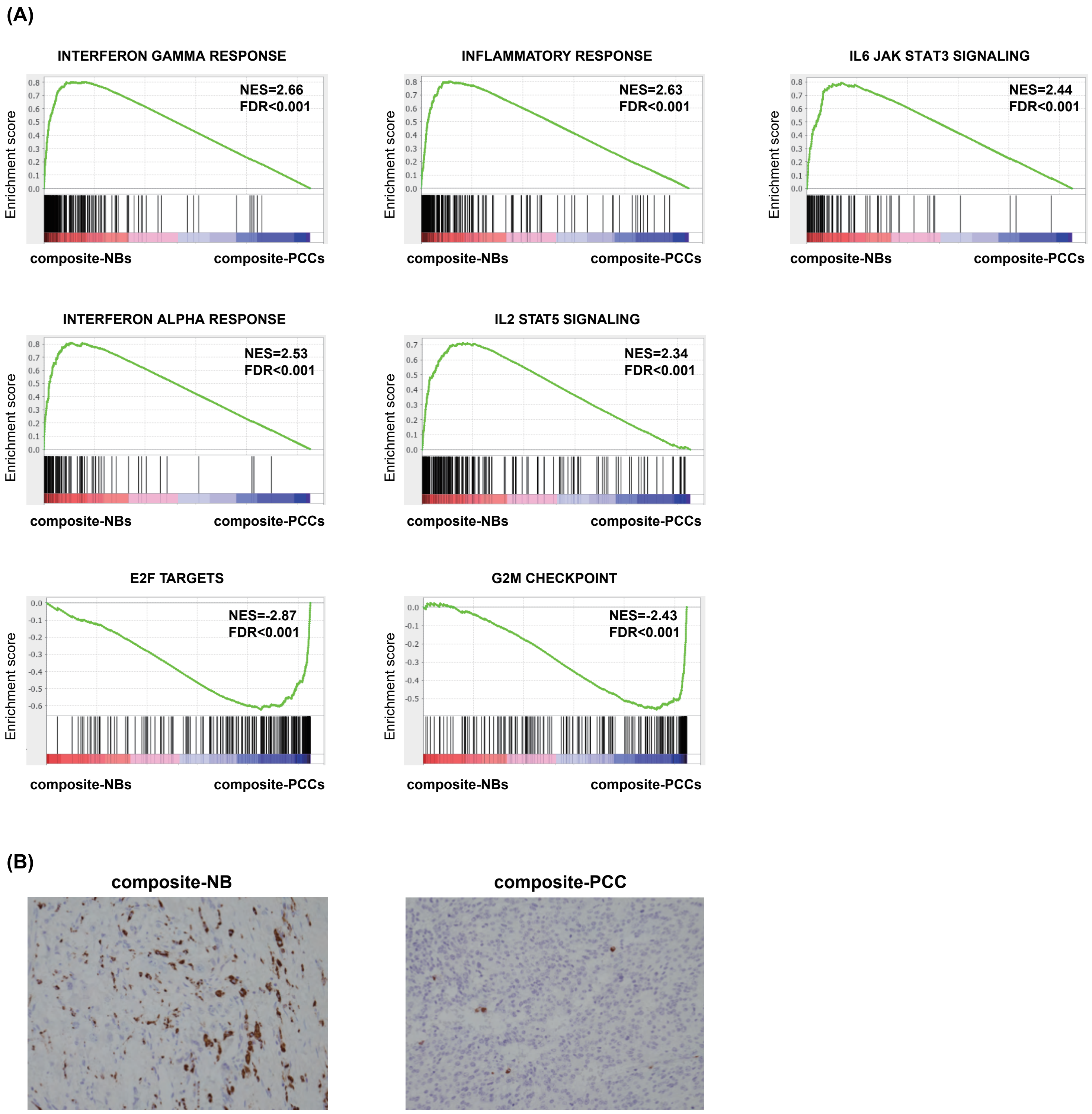

Figure S4. The enrichment plots for the most enriched Hallmark pathways, and Immunohistochemical staining of the composite-NB and PCC with the anti-CD68 monoclonal antibody. A, Gene sets of IFN-γ response, inflammatory response, IL-6 JAK STAT3 signaling, IFN-α response, IL-2 STAT5 signaling, E2F target, and G2M checkpoint are illustrated as enrichment plots. B, The NB component is more strongly positive for CD68 staining than PCC component (original magnification: 100×). NES, normalized enrichment score; FDR, false discovery rate

Figure S5

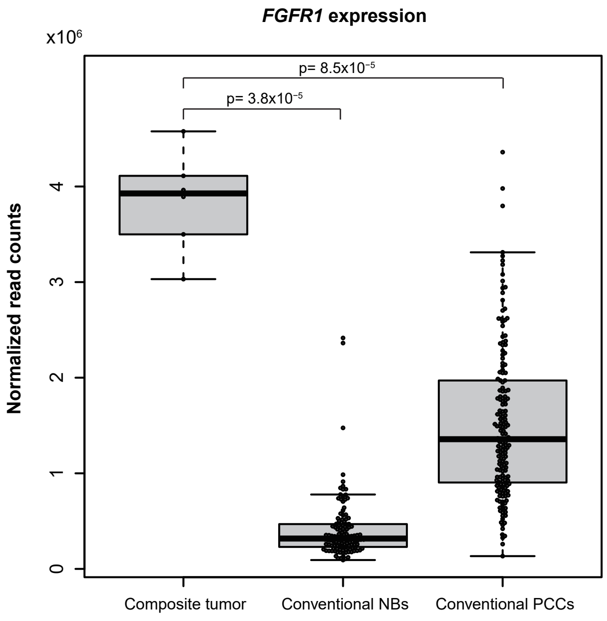

Figure S5. *FGFR1* expressions were elevated in composite tumor samples compared with conventional NBs (TARGET and DNA Data Bank of Japan cohorts 9) or PCCs (TCGA cohort). Statistical analyses were performed to compare normalized *FGFR1* expressions with the Wilcoxon rank-sum test. Normalized expression was calculated from the read counts with DEseq2.

Figure S6

Adrenal gland | **Intra-abdominal lymph node**

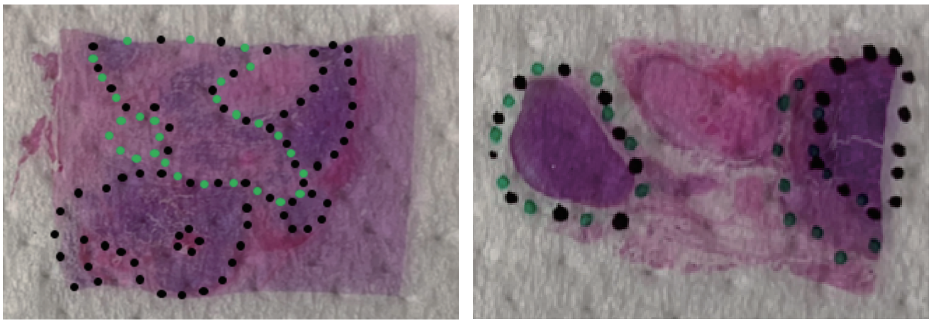

Green: **NB**
Black: **PCC**
Green and Black: **Mixed** (NB+PCC)

Green: **NB**
Black: **PCC**
Green and Black: **MiXed** (NB+PCC)

Figure S6. The NB, PCC, and mixed lesions in FFPE specimen. NB (green dots), PCC (black dots), and mixed (comprising NB and PCC components) (green/black dots) lesions are represented.

Figure S7

(A)

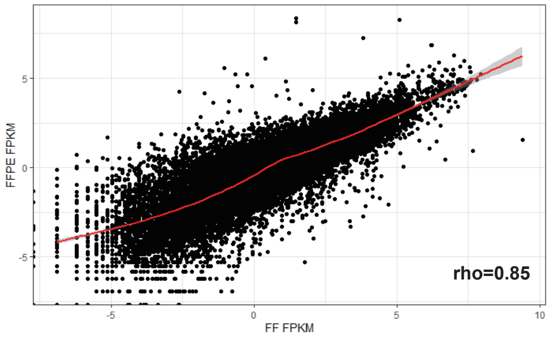

(B)

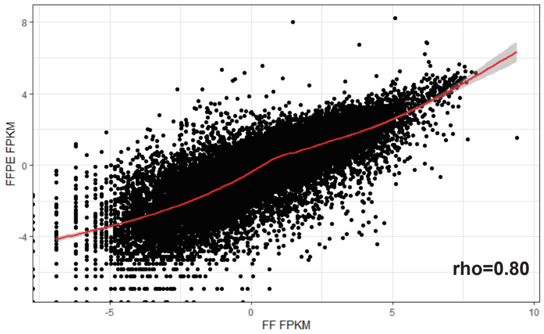

(C)

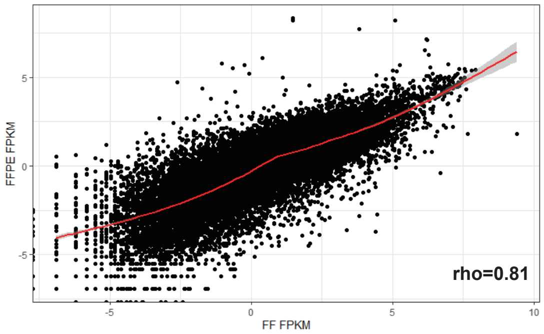

Figure S7. Based on fragments per kilobase of exon per million mapped reads, the comparison between a bulk sample of fresh frozen (FF) and NB component (A), PCC component (B), or mixed component (C) of FFPE sample in the metastatic intra-abdominal lymph node.
