# Supplementary material for: Oncogenic FGFR1 mutation and amplification in common cellular origin in a composite tumor with neuroblastoma and pheochromocytoma
Source: Cancer Sci. 2022 Feb 16;113(4):1535–41. doi: 10.1111/cas.15260 (PMC8990717; doi:10.1111/cas.15260)
Supplement: Supplementary file 3 — Appendix S1 [file CAS-113-1535-s002.docx]

**Supporting Information**

**MATERIALS AND METHODS**

**Patient samples**

We analyzed the primary adrenal gland and metastatic intra-abdominal lymph node samples from the fourth surgery. Genomic DNA and RNA were extracted from the NB, PCC, and mixed components of the FFPE samples of both organs (Figure S6). Additionally, the RNA from a fresh frozen sample of the metastatic intra-abdominal lymph node was used for quality control; moreover, matched normal renal tissues from FFPE samples were used as the germline control for WES and amplicon deep sequencing.

DNA and RNA were isolated from FFPE samples using a FormaPure XL Total kit (Beckman Coulter, Inc., Indianapolis, USA), and RNA was isolated from a fresh frozen sample using a RNeasy Mini Kit (Qiagen, Hilden, Germany).

**Immunohistochemistry analysis**

Immunohistochemical stains were performed on FFPE tissue sections (3μm), using Benchmark ULTRA system (Ventana Medical Systems, USA) and antibodies against the following antigens: Chromogranin A (clone LK2H10, ready to use, Biocare Medical, USA), PGP9.5 (clone 10A1, dilution, 1:40, Leica Biosystems, Germany), Neurofilament (clone 2F11, dilution, 1:50, DAKO, Denmark), CD68 (clone PG-M1, ready to use, DAKO).

**Whole-exome sequencing and mutation calling**

For WES, DNA libraries were prepared using an xGen Exome Research Panel (IDT, Coralville, USA). Enriched exome libraries were sequenced on an Illumina HiSeq X platform using the 150-bp paired-end mode. Our in-house Genomon v2.6.2 pipeline (<https://genomon.readthedocs.io/ja/latest/>) called the somatic variants with mapping quality score ≥ 20; base quality score ≥ 15; exonic/splice-site mutations; strand ratio not equal to 0/1; depths in both tumor and normal ≥ 8; number of variant reads in the tumor ≥ 4; VAFs ≥ 0.05 in tumor samples and < 0.05 in normal samples; EBCall, P ≤ 10^−3^ ^1^; and Fisher's exact test, P ≤ 10^−2^. Furthermore, visual inspection on the Integrative Genomics Viewer browser (<https://software.broadinstitute.org/software/igv/>) removed mapping errors.

Germline mutations were identified by assessing the WES data of the control were with VAFs > 0.2 and exclusion of the known variants in the 1000 Genomes Project (October 2014 release), National Heart, Lung, and Blood Institute, Exome Sequencing Project 5400, and the Human Genome Variation Database (February 2016 release).

**Validation of detected mutations**

PCR-based amplicon deep sequencing (depth ≥ 1 000 ×) validated candidate mutations using matched normal samples. The NotI restriction site was included in each primer as a linker sequence. Then, the amplified products were digested with NotI, ligated, fragmented, and used for deep sequencing library preparation ^2^. The candidate variants with sufficiently large VAFs in a tumor sample (≥ 0.01) that were at least 5 times that of the paired normal sample and the sequencing depth ≥ 1 000 were validated (Table S1).

**Phylogenetic analysis**

We used 108 validated mutations to construct a branch-based phylogenetic tree using the bootstrap resampling technique implemented in ClonEvol ^3^.

**Analysis of alterations in copy number**

The samples examined by WES were analyzed for genomic copy number using the signal data calculated from the sequence reads. The alternations in copy numbers were evaluated using our in-house CNACS pipeline (<https://github.com/papaemmelab/toil_cnacs>) ^4^. The depth was calculated from the weighted sum of the fragments accounting for length and GC biases during sequencing library amplification. The depths were compared with those of the pooled controls. Genomic copy number was estimated from the depth ratios using the circular binary segmentation method with the DNA copy package in R. The allele frequencies of the heterozygous SNPs assessed allelic imbalance covered by >50 reads.

**RNA sequencing and gene expression analysis**

Sequencing libraries from fresh frozen or FFPE samples were constructed using a NEBNext Poly(A) mRNA Magnetic Isolation Module or rRNA Depletion Kit v2, then an Ultra RNA Library Prep Kit for Illumina (New England Biolabs, Ipswich, Massachusetts, USA). The library was sequenced using an Illumina HiSeq X platform with a 150-bp paired-end read protocol. Sequence alignment and read counting were conducted using the Genomon v2.6.2 pipeline. Normalized count data obtained from the variance-stabilizing transformation function of the R package DESeq2 were used for clustering analysis. Cluster stability was ascertained via consensus clustering with 1 000 iterations using the R package ConsensusClusterPlus. Heat maps were generated with normalized count data using pheatmap 1.0.7. Gene set enrichment analysis was performed (version 4.0.3; <https://www.gsea-msigdb.org/gsea/index.jsp>).

The R package bseqsc v.1.0 (BSEQ-sc) ^5^ and CIBERSORTx ^6^ were used to deconvolute bulk RNA sequencing datasets of composite-NBs and TARGET cohort NBs with scRNA-seq-derived cell populations ^7^.

**Accuracy of RNA-seq data generated from FFPE samples**

We evaluated the accuracy of the RNA sequencing data from the NB, PCC, and mixed components of FFPE samples in the metastatic intra-abdominal lymph node than that of the fresh frozen samples of the same lesion. The FFPE (composite-NB lesion) and fresh frozen libraries had highly correlated fragments per kilobase of exon per million mapped reads measures (FPKM), with Spearman rho estimate calculated correlation 0.846 (Figure S7), consistent with the previous report that the protocol, with RNase H-based ribosomal RNA depletion, exhibited the least variability in gene expression measurements, the strongest correlation between fresh frozen and FFPE samples, and generally representative of the transcriptome from conventional fresh frozen RNA-seq protocols ^8^.

**REFERENCES**

1 Shiraishi Y, Sato Y, Chiba K, et al. An empirical Bayesian framework for somatic mutation detection from cancer genome sequencing data. *Nucleic Acids Res*. 2013; 41: e89.

2 Yoshida K, Sanada M, Shiraishi Y, et al. Frequent pathway mutations of splicing machinery in myelodysplasia. *Nature*. 2011; 478: 64-69.

3 Dang HX, White BS, Foltz SM, et al. ClonEvol: clonal ordering and visualization in cancer sequencing. *Ann Oncol*. 2017; 28: 3076-3082.

4 Yoshizato T, Nannya Y, Atsuta Y, et al. Genetic abnormalities in myelodysplasia and secondary acute myeloid leukemia: impact on outcome of stem cell transplantation. *Blood*. 2017; 129: 2347-2358.

5 Baron M, Veres A, Wolock SL, et al. A Single-Cell Transcriptomic Map of the Human and Mouse Pancreas Reveals Inter- and Intra-cell Population Structure. *Cell Syst*. 2016; 3: 346-360.e344.

6 Newman AM, Steen CB, Liu CL, et al. Determining cell type abundance and expression from bulk tissues with digital cytometry. *Nat Biotechnol*. 2019; 37: 773-782.

7 Jansky S, Sharma AK, Körber V, et al. Single-cell transcriptomic analyses provide insights into the developmental origins of neuroblastoma. *Nat Genet*. 2021.

8 Li J, Fu C, Speed TP, Wang W, Symmans WF. Accurate RNA Sequencing From Formalin-Fixed Cancer Tissue To Represent High-Quality Transcriptome From Frozen Tissue. *JCO Precis Oncol*. 2018; 2018.

9 Kimura S, Sekiguchi M, Watanabe K, et al. Association of high-risk neuroblastoma classification based on expression profiles with differentiation and metabolism. *PLoS One*. 2021; 16: e0245526.

**Legends for Supplementary Figures**

**Figure S1.** Copy number plots of each of the six samples of the composite tumor.

(A) NB lesion in the primary adrenal gland

(B) PCC lesion in the primary adrenal gland

(C) Mixed lesion in the primary adrenal gland

(D) NB lesion in the metastatic intra-abdominal lymph node

(E) PCC lesion in the metastatic intra-abdominal lymph node

(F) Mixed lesion in the metastatic intra-abdominal lymph node

**Figure S2.** Consensus clustering analysis of the six samples of the composite tumor.

(A) CDF plots and consensus matrices for consensus clustering (n = 1,000) of the six samples of the composite tumor. Two thousand differentially expressed genes were selected.

(B) Unsupervised consensus clustering of the six samples of composite tumor identified two distinct clusters corresponding to histopathological features of NB and PCC. NB, neuroblastoma; PCC, pheochromocytoma; mixed, mixed components of NB and PCC without clear boundaries.

**Figure S3.** The composition of composite-NBs and PCCs, and the gene expression profiles in composite-PCCs and 173 TCGA PCC/PGL samples based on unsupervised consensus clustering.

(A) Composition of composite-NBs and PCCs based on deconvolution of bulk RNA sequencing data with fetal adrenal cell populations using BSEQ-sc.

(B) CDF plots and consensus matrices for consensus clustering (n = 1,000) of two composite-PCCs and 173 TCGA PCC/PGL samples. Five hundred differentially expressed genes were selected.

(C) A heatmap of the expression data of two composite-PCCs and 173 TCGA PCC/PGL samples. Composite-PCCs were grouped into the same cluster of kinase signaling of TCGA samples.

**Figure S4.** The enrichment plots for the most enriched Hallmark pathways, and Immunohistochemical staining of the composite-NB and PCC with the anti-CD68 monoclonal antibody.

(A) Gene sets of IFN-γ response, inflammatory response, IL-6 JAK STAT3 signaling, IFN-α response, IL-2 STAT5 signaling, E2F target, and G2M checkpoint are illustrated as enrichment plots. NES, normalized enrichment score; FDR, false discovery rate

(B) The NB component is more strongly positive for CD68 staining than PCC component (original magnification: 100×).

**Figure S5.** *FGFR1* expressions were elevated in composite tumor samples compared with conventional NBs (TARGET and DNA Data Bank of Japan cohorts ^9^) or PCCs (TCGA cohort).

Statistical analyses were performed to compare normalized *FGFR1* expressions with the Wilcoxon rank-sum test. Normalized expression was calculated from the read counts with DEseq2.

**Figure S6.** The NB, PCC, and mixed lesions in FFPE specimen.

NB (green dots), PCC (black dots), and mixed (comprising NB and PCC components) (green/black dots) lesions are represented.

**Figure S7.** Based on fragments per kilobase of exon per million mapped reads, the comparison between a bulk sample of fresh frozen (FF) and NB component (A), PCC component (B), or mixed component (C) of FFPE sample in the metastatic intra-abdominal lymph node.

**Table S1.** Somatic mutations detected by whole-exome sequencing and validated by amplicon deep sequencing in six samples.

**Table S2.** Marker genes employed for deconvolution of RNA sequencing data of composite-NBs and TARGET cohort NBs.
